# Involvement of MCH-oxytocin neural relay within the hypothalamus in murine nursing behavior

**DOI:** 10.1038/s41598-021-82773-5

**Published:** 2021-02-08

**Authors:** Yoko Kato, Harumi Katsumata, Ayumu Inutsuka, Akihiro Yamanaka, Tatsushi Onaka, Shiro Minami, Chitose Orikasa

**Affiliations:** 1 Department of Bioregulation, Institute for Advanced Medical Science, Nippon Medical School, Kawasaki, 211-8533 Japan; 2 Department of Physiology, Jichi Medical University, Shimotsuke, Tochigi 329-0498 Japan; 3 Department of Neuroscience II, Research Institute of Environmental Medicine, Nagoya University, Nagoya, 464-8601 Japan

**Keywords:** Neuroscience, Physiology, Environmental social sciences, Endocrinology

## Abstract

Multiple sequential actions, performed during parental behaviors, are essential elements of reproduction in mammalian species. We showed that neurons expressing melanin concentrating hormone (MCH) in the lateral hypothalamic area (LHA) are more active in rodents of both sexes when exhibiting parental nursing behavior. Genetic ablation of the LHA-MCH neurons impaired maternal nursing. The post-birth survival rate was lower in pups born to female mice with congenitally ablated MCH neurons under control of tet-off system, exhibiting reduced crouching behavior. Virgin female and male mice with ablated MCH neurons were less interested in pups and maternal care. Chemogenetic and optogenetic stimulation of LHA-MCH neurons induced parental nursing in virgin female and male mice. LHA-MCH GABAergic neurons project fibres to the paraventricular hypothalamic nucleus (PVN) neurons. Optogenetic stimulation of PVN induces nursing crouching behavior along with increasing plasma oxytocin levels. The hypothalamic MCH neural relays play important functional roles in parental nursing behavior in female and male mice.

## Introduction

Regulation of the neural circuits that govern social behavior, including parental behavior, is a topic of immense interest in neuroscience. After parturition, females exhibit maternal care, and at the same time, maternal care exhibited by virgin females is easily activated by priming with exposure to pups^1^. However, virgin males that commit infanticide have inactive neural circuits governing parental behaviors, whereas males that have experienced mating with gestating females exhibit parental behavior^2,3^. In our previous studies conducted virgin animals, we observed that the intrinsic features of parental behavior characteristics were easily elicited in both sexes after social isolation^4^. It has been reported that the preoptic area^5–9^ and the anteroventral periventricular nucleus (AVPV)^10^ influence parental behavior, which was also observed in studies conducted using virgin animals. A subpopulation of galanin-expressing neurons was found to be activated during parenting episodes involving pup grooming and retrieving^6^. Neuronal connections from the AVPV to the paraventricular hypothalamic nucleus (PVN) were found to influence oxytocin secretion^10^. Oxytocin-secreting neurons play a crucial role in the onset and maintenance of maternal behavior in rodents^10–12^. Further evidence showed that PVN neurons project anatomically posterior to the lateral hypothalamic area (LHA)^13^ to regulate the level of melanin concentrating hormone (MCH)^14^, a neuromodulator that integrates physiological functions^15–23^. Abolishing the expression of MCH will decrease maternal behaviors, especially in decreasing litter sizes^24^. Although these observations suggest that MCH neurons mediate or modulate parental nursing behaviors, further research is needed to determine how MCH neurons are projected through a detailed investigation on parental behavior regulation.

## Results

### MCH enables pup nursing behavior

**Figure 1:**
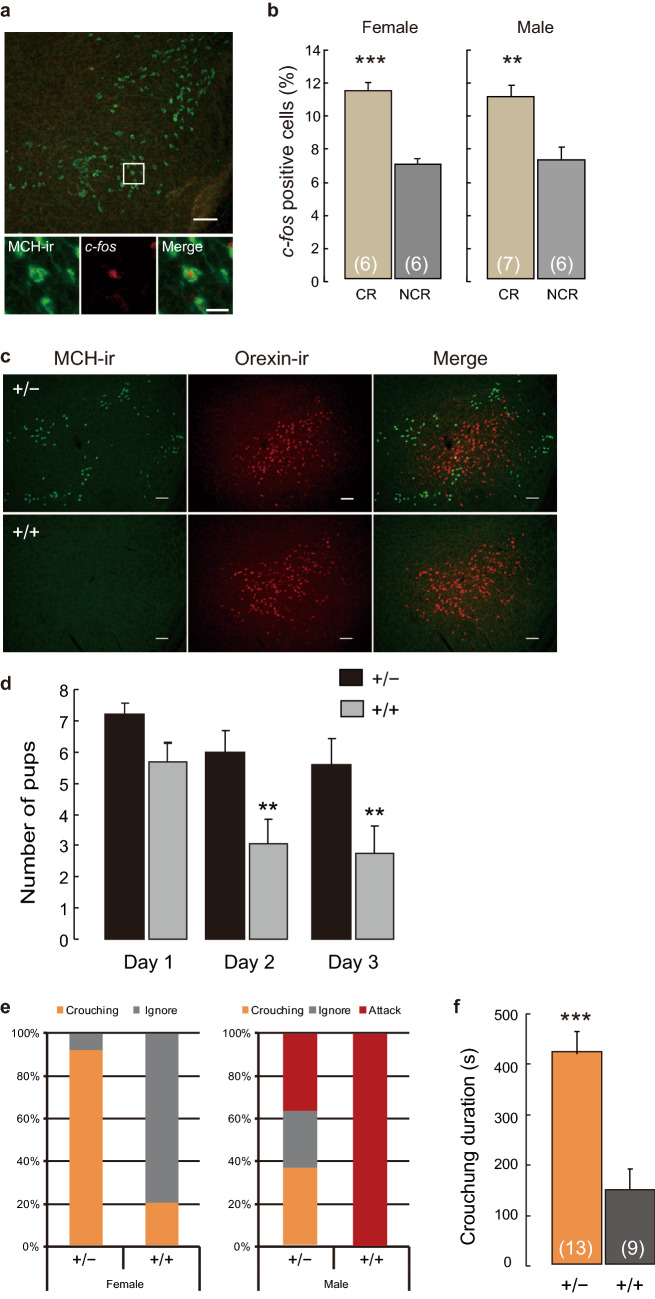
Parental nursing behaviour activates MCH neuron in the LHA and cell-specific ablation of MCH neurons impairs nursing behaviour in both virgin female and male mice. (**a**) Simultaneous visualisation of *c-fos*- and MCH-immunoreactive (ir) cells in coronal brain sections at the level of the LHA. Scale bars are 100 µm in the above and 20 µm in the bottom; a square indicates place of bottom figures in the above. (**b**) Quantitative analysis of *c-fos*-ir in MCH neurons of female (left) and male (right) ddN mice that performed crouching (CR) or did not perform crouching (NCR). Mean ± S.E.M. t-test, ***p < 0.001, **p < 0.01. (**c**) Ablation of MCH neurons in *MCH-tTA; TetO DTA* bigenic mice. MCH neurons and orexin neurons in *MCH-tTA; TetO DTA* bigenic +/− (top) and +/+ (bottom) mice. Scale bar, 100 μm. (**d**) Survival rate of pups after parturition of *MCH-tTA; TetO DTA* bigenic +/+ and +/− female mice. Two-way ANOVA, day after parturition F(2, 72) = 5.58, **p = 0.006, Genotype F(1, 72) = 16.6, ***p < 0.0001, post-hoc test with Bonferroni, +/− vs +/+ , Day 1, *p* = 0.14, non-significance, Day 2, **p = 0.006, Day 3, **p = 0.008. (**e**) Percentage of parental behaviour or attack pups of virgin bigenic +/+ and +/− females (left) and males (right). The ratio of parental behaviour (crouching) in females was analysed by Chi-squared test, X-squared = 9.56, df = 1, *p* = 0.002 **. (**f**) Duration of crouching behaviour of virgin *MCH-tTA; TetO DTA* bigenic +/+ female mice when compared with +/− females. Mean ± S.E.M. t-test, ***p < 0.001.

In this study, we first examined the expression of the immediate early gene *c-fos* in the MCH neurons of ddN mice during maternal nursing behavior. All experimental groups were prepped after social isolation to readily derive the conditions of parenting behavior^4^. We observed significantly higher *c-fos* expression in the MCH neurons of the virgin female and male mice that showed nursing crouching behavior compared with mice that ignored their pups (Fig. 1a,b). To determine the neural basis of nursing crouching behavior, we used genetically ablated cell-specific MCH neurons to create innate MCH-neuron knockout animals *MCH-tTA; TetO diphtheria toxin A fragment (DTA)* bigenic mice^18^. Cell-specific ablation of MCH neurons without abolishing orexin neurons was observed (Fig. 1c) in the *MCH-tTA; TetO DTA* bigenic (+/+) mice. The pup survival rate after parturition in females with ablated MCH neurons was lower than that in bigenic (+/−) controls (Fig. 1d). We also examined the maternal behavior of virgin female and male +/+ bigenic mice. The ablation of MCH neurons did not affect the oestrus cycle (Supplementary Fig. 1a–c), reproductive success (Supplementary Fig. 1d,e), whereas the body weight of +/+ bigenic mice was significantly lower in both sexes (Supplementary Fig. 2) in accordance with a previous study^25^. Compared with the +/− controls, virgin females with ablated MCH neurons were less interested in pups and maternal care similar to +/+ bigenic mothers (Fig. 1e). The virgin +/+ bigenic females showed significantly lowered crouching than +/− bigenic controls (X^2^ = 11.29, df = 1, *p* = 0.001) (Fig. 1e); however, no significant difference was observed in retrieving behavior (X^2^ = 1.69, df = 1, *p* = 0.193) (Table 1). The duration of crouching in +/+ bigenic female was significantly shorter than that of +/− controls (Fig. 1f). In contrast, virgin +/+ bigenic males abolishing MCH neurons were more aggressive toward their pups (Fig. 1e) and intruder males than the +/− controls (Supplementary Fig. 3). These results suggest that the effect on the ablation of MCH neurons was slightly different in females and males. The results for bigenic male partially agreed with the findings reported in a previous study^24^. However, in females, we found that abolishing MCH neurons affected maternal crouching behavior (Fig. 1e,f). Our data suggest that MCH neurons play a role in neuromodulating functions of parental nursing behavior in mice.

**Table 1:** Number and percentage of *MCH-tTA; TetO DTA* bigenic females in each response category.

Category		MCH-tTA; TetO DTA bigenic females			
		+/+ (9)		+/− (13)	
		Number	%	Number	%
Retrieving		3	33.3	8	61.5
Crouching		2	22.2**	12	92.4

**p < 0.01, Chi-square test with Bonferroni post hoc analysis, different from +/− females.

### Decreasing nursing crouching behavior by MCH neuron-specific ablation

**Figure 2:**
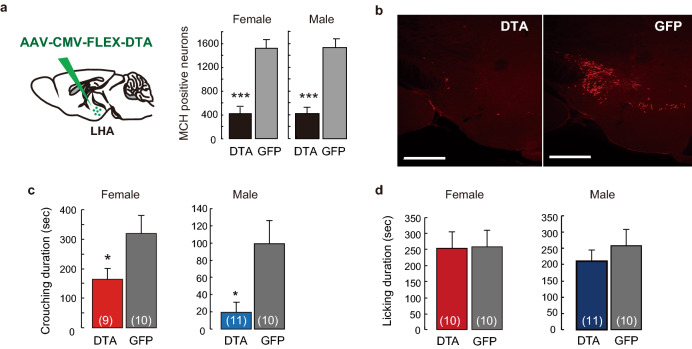
Cre-dependent ablation of MCH-producing neurons effects of parenting nursing behaviour. (**a**) Cre-MCH dependent expression with AAV-CMV-FLEX-DTA injection affected the number of MCH positive neurons specifically in the LHA in both sexes. (**b**) An example of ablation by AAV-DTA injection. Scale bars, 500 μm. (**c**,**d**) Effect of AAV-DTA injections on the duration of crouching (**c**) and licking (**d**). Mean ± S.E.M. t-test, *p < 0.05, ***p < 0.001.

Consequently, we focused on maternal nursing behavior to elucidate whether these neurons are involved in nursing crouching using *MCH-Cre* mice. We performed Cre-dependent DTA ablation of MCH neuron, using adeno-associated virus (AAV)-DTA (Fig. 2a) in virgin mice, abolishing MCH-expressing neurons in 72.4% ± 7.1% of females and 73.9% ± 6.2% of males (Fig. 2a,b) without abolishing orexin neurons (Supplementary Fig. 4a). In virgin *MCH-Cre* mice injected with AAV-green florescent protein (GFP), crouching was slightly facilitated after social isolation (Fig. 2c). Virgin females and males that lacked MCH-expressing neurons (by AAV-DTA) spent significantly less time exhibiting crouching behavior than those of AAV-GFP control (Fig. 2c); licking (Fig. 2d) and locomotor activity (Supplementary Fig. 4b) were unaffected. This finding indicates that MCH neurons are involved in the regulation of parental nursing in both females and males.

### Increasing nursing couching behavior by chemogenetics and optogenetic activation of MCH neurons

**Figure 3:**
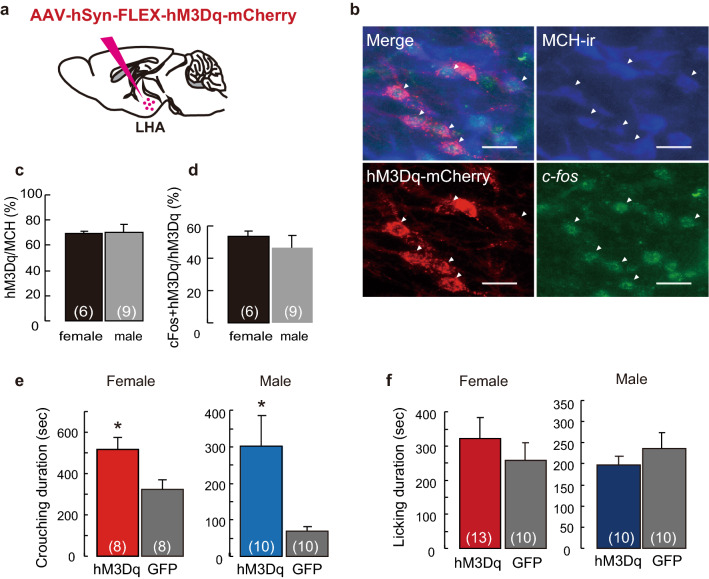
Cre-dependent activation of MCH-producing neurons effects of parenting and transduction efficiency of hM3Dq-mCherry AAV vector. (**a**) Illustration of sagittal brain sections injected with AAV-hSyn-FLEX-hM3Dq-mCherry into the LHA. (**b**) Simultaneous visualisation of Cre-MCH-dependent expression of hM3Dq-mCherry and *c-fos* positive-MCH-ir cells (arrowheads). Scale bars, 20 μm. (**c**) Quantitative analysis of MCH neurons injected by hM3Dq-mCherry AAV vector in MCH-Cre female and male mice. Transduction efficiencies were calculated in both sexes by injecting hM3Dq-mCherry AAV vector in MCH neurons. (**d**) The percentage of *c-fos* expression in MCH neurons injected with the AAV-hM3Dq-mCherry in MCH-Cre mice. (**e**) Duration of crouching and (**f**) licking from the pup presentation, 90 min after CNO injections. Mean ± S.E.M. t-test, *p < 0.05.

**Figure 4:**
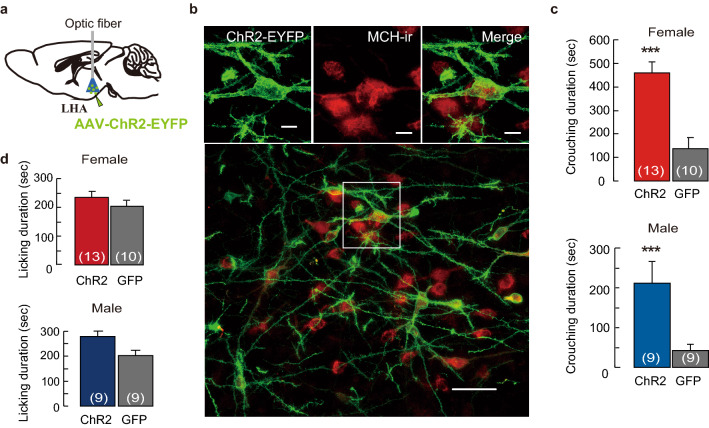
Optogenetic activation of MCH neurons prompts parental nursing behaviour in the LHA. (**a**) Illustration of sagittal brain sections injected with AAV-EF1a-DIO-hChR2 (E123T/T159C)-EYFP (ChR2-EYFP) and optogenetic stimulation in the LHA. (**b**) Co-labelled MCH-ir (red) and ChR2-EYFP (green) cells in the LHA. Scale bars are 10 µm in the above and 50 µm in the bottom; a square indicates place of above figures in the bottom. LHA-MCH neurons expressed ChR2-EYFP with considerable frequency. (**c**) Crouching and (**d**) licking duration of optogenetic stimulation in the LHA. Mean ± S.E.M. t-test, ***p < 0.001.

Using chemogenetics, we further examined the involvement of MCH neurons, which are activated by the synthetic ligand clozapine-N-oxide (CNO)^26^ in *MCH-Cre* mice injected with the AAV-hM3Dq-mCherry into the LHA (Fig. 3a). We found that 69.7% ± 1.9% of MCH neurons in females and 70.3% ± 6.8% in males expressed hM3Dq-mCherry (Fig. 3b,c) and c-*fos* (Fig. 3b,d). From the pup presentation test that began 90 min after the CNO injection, the crouching duration significantly increased in virgin *MCH-Cre* females and males compared with that of AAV- GFP controls (Fig. 3e). No significant difference in licking behavior (Fig. 3f) and locomotor activity (Supplementary Fig. 4c) was observed. *c-fos* expression was 53.4% ± 3.3% of hM3Dq-expressing MCH neurons in females and 46.7% ± 7.3% of these in males (Fig. 3d). MCH neurons have been suggested to be important for controlling nursing crouching behavior in both virgin females and males. To confirm whether another method of MCH-neuron activation induces nursing behavior, we performed optogenetic simulation of MCH neurons (Fig. 4a). An in vitro study was performed to confirm the stimulation of channelrodopsin 2 (ChR2) expressing cells in our (Supplementary Fig. 5a) and previous studies^23^. ChR2-enhanced yellow fluorescent protein (EYFP) was selectively expressed on MCH-producing neurons (Fig. 4b and Supplementary Fig. 5a) in the LHA of virgin *MCH-Cre* females and males. Before the behavioral test, we bilaterally inserted optic fibres just above the LHA (Fig. 4a). Stimulation through the optic fibres was provided to ChR2 expressing mice and control GFP mice equally during the pup presentation test. Initially, blue light pulses (473 nm, 15 mW, 10 ms, 10 Hz) were applied. No parental behavior was observed, while the mice stayed still away from or next to the pups. This blue laser pulses (475 ± 17.5 nm, 2.5 mW,10 ms, 10 Hz at the tip of the fibre cannula) was similar to the condition of REM sleep induction which is previously reported in *MCH-tTA; TetO* ChR2 mice^18^. We determined the appropriate blue laser pulses (473 nm, 10 ms, 0.5 Hz, 1 mW at the tip of the fibre cannula) to be applied through the optic fibre (COM2-DF2-500, LUCIR Inc.) during the test sessions. ChR2-EYFP was expressed in almost 40% of MCH-producing neurons (Supplementary Fig. 5b). Pulse photo-stimulations of 1 mW (0.5 Hz) pulse to MCH neurons infused with ChR2 significantly increased crouching behavior (Fig. 4c) but did not affect licking behavior (Fig. 4d) in females and males. We observed that the condition of optogenetic photo-stimulation required differed between males and females; photo-stimulations were started 5 min before pup presentation in males but not in females. This difference may be caused by unknown sex-dependent differences in neural circuits. During optogenetic stimulation of the LHA, which induces crouching behavior (Fig. 4c), *c-fos*-expressing cells were visualised in ChR2-EYFP-positive MCH neurons (Supplementary Fig. 5a,c). Photo-stimulation did not affect locomotion in the LHA (Supplementary Fig. 6). These results also indicate that the MCH neurons are cardinal in the regulation of parental nursing behavior.

### MCH-oxytocin neuronal relay modulates nursing behavior

**Figure 5:**
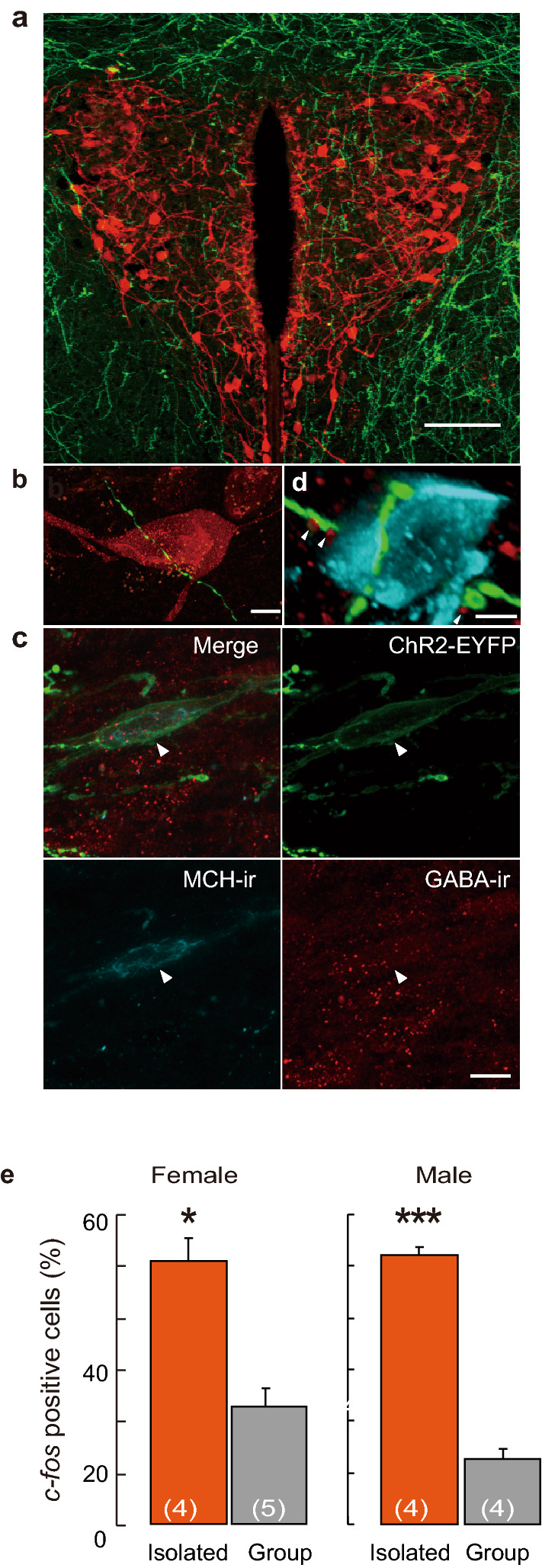
ChR2-EYFP fibres from LHA-MCH neurons project into PVN-oxytocin neurons. (**a**) ChR2-EYFP fibre from MCH neurons into the PVN. ChR2-EYFP fibre surrundings and into the PVN. Scale bar, 100 µm. (**b**) ChR2-EYFP fibre (green) identified in close vicinity of the oxytocin neurons (red). Scale bars, 5 µm. (**c**) Simultanous visualization of GABA-ir and ChR2-EYFP in LHA-MCH neuron (arrowheads). Photopmicrograph revealing ChR2-EYFP (green) expressing MCH-ir (blue) neuron containing GABA (red) in the LHA. Scale, 10 µm. (**d**) GAD65-ir punctate (red) deposits (arrowhesds) in apposition with MCH-ChR2 fiber (green) adjacent to the oxytocin neuron (blue) of the PVN. Scale bar, 5 µm. (**e**) Percentage of *c-fos* positive oxytcin neurons of isolated or group-housing female and male mice after muscimol (10 ng) injection into the PVN. The animals were perfused 90 min after the injection. Mean ± S.E.M. t-test, *p < 0.05, ***p < 0.001.

Using Cre-dependent ChR2-EYFP, we observed fluorescently labelled fibres projecting from LHA-MCH to the PVN in both females and males (Fig. 5a). Projection analysis also showed that LHA-MCH neurons reached several brain regions in both sexes (Supplementary Fig. 7) including the diagonal band broca, lateral septum and lateral preoptic area. The dense MCH neuronal EYFP fibres expressing ChR2 derived from LHA-MCH neurons projected close proximity to the oxytocin neurons of the PVN (Fig. 5a,b). MCH neurons synthesise and could utilise GABA as a neurotransmitter. GABA neurons innervate neurosecretory somata and dendrites^27,28^ and as well as GABAergic synapses formed to oxytocinergic neurons^28^. We confirmed that the ChR2-expressing MCH neurons contained GABA (Fig. 5c). Moreover, we detected GAD 65-ir puncta in ChR2-EYFP fibres (Fig. 5d). GABAergic projection derived from MCH neuron could regulate the PVN-oxytocin neurons. We stereotacxically injected GABA agonist, musicimol into the PVN. We found significantly increased *c-fos* expressing oxytocin neurons in the socially isolated females and males of the PVN than in those of the group-housing female and male mice (Fig. 5e), indicating that the possible involvement of MCH neuronal innervation is excitatory.

**Figure 6:**
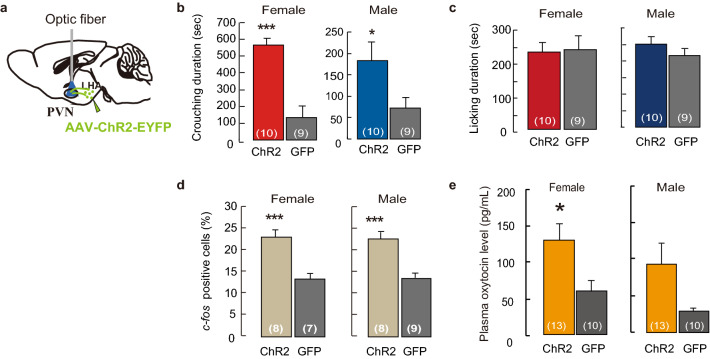
Optogenetic activation of MCH ChR2-expressing fibres prompts parental nursing behaviour in the PVN. (**a**) Illustration of optogenetic stimulation in the PVN with ChR2-EYFP fibre from MCH neurons. (**b**) Duration of crouching and (**c**) licking during optogenetic stimulation in the PVN. (**d**) The percentage *c-fos* positive oxytocin neurons in which ChR2 -expressed fibres were stimulated in the PVN. (**e**) Oxytocin levels in plasma samples after optogenetic stimulation in the PVN. Mean ± S.E.M. t-test, ***p < 0.001, *p < 0.05.

The PVN neurons secrete oxytocin, which contributes to maternal behavior in mice^11,12^. We then examined whether MCH neurons projecting from the LHA to the PVN were involved in the regulation of crouching behavior. The optic fibres placed just above the PVN (Fig. 6a) were used to perform photo-stimulation using the same procedure of investigating LHA. During the pup presentation test, both females and males exhibited crouching behavior of longer duration than that of the controls (Fig. 6b) but did not affect licking behavior (Fig. 6c) or locomotion (Supplementary Fig. 6). We observed significantly higher *c-fos* expression in the oxytocin neurons in the PVN of the photo-stimulation virgin female and male mice (Fig. 6d). We collected plasma samples immediately after pup presentation to measure oxytocin levels with PVN photo-stimulation, which were significantly higher in ChR2 than those in GFP females (Fig. 6e). Males showed the same trend, however, the differences between the ChR2 and GFP groups were not significant (*p* = 0.057).

## Discussion

We elucidated that the tuned activity of MCH is entailed for nursing associated with social interactions. It also modulates MCH neural circuit characteristics onto oxytocin neurons in the PVN. The results demonstrated that the relay between LHA and PVN is important in the regulation of parenting nursing behavior in both sexes. Furthermore, increased plasma oxytocin levels after photo-simulation to the ChR2-expressed fibre in the PVN comprise the modulation of social nursing interaction.

Although optogenetic conditions were virtually applied under exactly the same condition of induced REM sleep^18^, no parental behavior was observed. We determined the appropriate blue laser pulses that are of lower frequency than that of REM sleep induction. This appears to be an alternative usage of an identical neurotransmitter in different types of behavior, which argues the multiple functions of MCH neurons in the brain. The changes in neuronal excitability dependent on the behavior state^29^ extend our results, prompting inherent behavior characteristics. The MCH-immunoreactive fibre (Fig. 5a,b) and its receptors are distributed in the PVN^30^. Although there exists much controversy regarding MCH neuronal transmitters, the MCH neurons in the LHA contain and release GABA^27^. The GABA-ir in the LHA-MCH neuron somata and GABAergic projections in the PVN were demonstrated in this study. Furthermore, the effect of the GABA agonist, muscimol resulted in the consequent increase of *c-fos* immunoreactivity in oxytocin neurons in the PVN in a mode of social isolation. The LHA-MCH GABAergic innervation into oxytocin neurons in the PVN can be considered to be excitatory. Although GABA predominantly acts as an inhibitory neurotransmitter of the brain, there exists an excitatory GABAergic activity in the MCH neurons during development^31^. However, extensive evidence of the excitatory action of GABA under stress or crucial physiological conditions in mature neurons has been reported^32–35^. Social isolation can be assumed as a favourable stressor in rodents^36^ and prompts changing the synaptic organisation action in the brain^37^. Therefore, social isolation stress may change the mode of GABAergic excitation.

The relay of this LHA neural projection to the PVN demonstrated in the present study and anatomical neural circuit from the PVN to LHA reported previously^14^, may contribute to the continued nursing crouching behavior. The LHA-PVN neural circuit is involved in the recurrent behavior^22^ by possibly including continuous crouching for completing lactation. On one hand, the periaqueductal gray (PAG) in the midbrain is responsible for reproduction, i.e., lordosis^38^ in sexual behavior and the maternal arched back crouching posture; on the other hand, no effect was observed on retrieval or in pup grooming in the female rat^39^. In contrast, the stimulation of galanin-expressing neurons in the medial preoptic area to the PAG projection increased the pup grooming behavior, but there was no effect on crouching in both female and male mice^8^. Alternatively, these maternal behaviors may be controlled and modulated by several neuronal inputs into the PAG from other brain regions. Although the MCH receptor in the PAG has not been confirmed^30^, it is necessary to define the possible involvement in neural circuits with neurotransmitter-derived LHA-MCH neurons projecting into the PAG in terms of motor control, which may prompt the maternal crouching behavior.

Oxytocin has been implicated in social reward^40^, and the involvement of PVN-LHA and LHA-PVN neural relay provides a rationale for explaining the continued nursing crouching behavior that sometimes persisted for a prolonged time period. Projections from the medial preoptic area to other functional brain areas have been reported to underlie parental behavior, excluding crouching behavior^8^. It is important to understand how the neural circuits that govern the social interaction of each behavior are induced or altered by social cues. Neural circuits involved in the heterogeneous components of this parental behavior have been extensively investigated at the levels of the brain area and neuromolecular materials^5,6,8,10,11,41^. It is possible that the centres governing distinct types of parental behaviors, e.g., retrieving and crouching are present in a variety of brain regions and are controlled independently. The implications of this diversity are not completely understood, thus necessitation further study to elucidate the neural integration and collaboration of each behavior involved in nursing and other parenting behaviors in mice. Each brain area and neuronal peptide must collaborate with and consolidate the multiple behavioral processes regulated by several components, resulting in forming parenting. The behavior centre presumed to be an integrative role in each social component of parenting behavior regulated by distinct brain regions, including diverse neuromolecular materials remains to be elucidated.

For protecting their offspring, mice have several neuronal peptides that organise the neural network for assuring maternal care. The MCH receptor is expressed throughout the reward circuitry, including the nucleus accumbens, for their consistency with oxytocinergic projection of the mesolimbic system^42^ and possibly involved in an emotional reinforcement of rewards. Therefore, the MCH neural networks in conjunction with oxytocin signalling that are required for social rewards facilitate pup survival and lead to a better chance of progeny persistence.

## Methods

### Animals

The experiments and animal housing were in accordance with the guidelines for the Care and Use of Laboratory Animals of Nippon Medical School following National Institutes of Health guidelines for the care and use of experimental animals and were approved by the Committee for Experimental Animal Ethics at Nippon Medical School. Mice were housed in 19 × 27 × 15 cm polypropylene cages with clean wood chip bedding. After weaning at 21 days of age, the mice were housed in our animal facility under controlled inversion illumination (lights on from 11 p.m. to 11 a.m.), temperature (23 °C) and humidity (50.0 ± 10%). Food and water were provided ad libitum*.* The inbred strain ddN mice were purchased from the RIKEN Bio-Resource Research Centre (Wako, Japan) and bred in our laboratory. The *MCH-Cre* bacterial artificial chromosome (BAC) transgenic line [STOCK Tg(Pmch-cre)1Lowl/J, 014099, Jackson Laboratory, Sacramento, CA, USA] was generated by insertion of the murine pro-melanin-concentrating hormone promoter/enhancer regions into the BAC transgene for controlling Cre-recombinase expression. To obtain complete congenital ablation of LHA-MCH neurons, we used the bigenic *MCH-tTA*^18^*; TetO DTA* [B6.Cg-Tg(*tetO-DTA*)1Gfi/J, 008468] (Jackson Laboratory).

### Viral injections

The AAV Helper-Free System (Agilent Technologies, Inc., Santa Clara, CA, USA) was used to produce AAV and purify the AAV vectors^43^. The plasmids pAAV-hSyn-FLEX-hM3Dq-mCherry, pAAV-hSyn-FLEX-mCherry and pAAV-EF1α-DIO-hChR2(E123T/T159C)-EYFP were purchased from Addgene (ID: 44361, 44362, 50459, 35509). pAAV-CMV-FLEX-hrGFP was constructed starting with the pAAV-hrGFP plasmid (Agilent Technologies), and pAAV-CMV-FLEX-DTA was constructed starting with the pAAV-MCS plasmid (Agilent Technologies), and its cell-specific ablation was confirmed as previously reported^44^. The injection volume was 600 nl per side.

### Behavioral tests

All the experiments began 2 h after the start of the dark phase and were video recorded (Sony HDR-cx670 HANDYCAM) under weak light conditions.

### Parental behavior

Each experimental female and male was placed in a clean cage with fresh bedding 2 days before testing followed by individual housing for approximately 1 week for females and 3 weeks for males. All behavioral tests were conducted during the day, 2–4 h after the lights were turned off. The observation began with the placement of three pups aged 4–7 days, in the corner opposite and farthest from the animals or the resident mouse’s nest. Parental behavior was assessed as previously reported^4^. Parental nursing behavior was assessed by time spent crouching, which was defined as the limb extension and the assuming of nursing-like posture over the pups. Time spent licking was also recorded. ‘Attack’ was defined as biting a pup, often accompanied by observable wounds on the pup, and ‘Ignore’ was defined as showing no response to the pups. If the pups were attacked, they were quickly removed, and the test was terminated. Behavioral data of mice that attacked pups were excluded from analysis of maternal behavior.

### Oestrus cycle observations in female bigenic mice

After sexual maturation at the age of 8 weeks, the oestrus cycle of female *MCH-tTA; TetO DTA* bigenic (+/+) and control mice (+/−) was assessed once daily for 19 days. Vaginal smears were observed under a light microscope to determine the oestrous cycle stages.

### Mating behavior

Each female *MCH-tTA; TetO DTA* bigenic bigenic (+/+) and control (+/−) mouse was introduced to a WT male on the day of proestrus. The session lasted 30 min starting between 5 p.m. and 6:30 p.m. Sexually experienced WT males were randomly assigned to the bigenic females. When successful mating behavior was observed, the female was kept with the WT male after the session. If mating behavior did not occur during the session, another male was introduced on the next oestrus day.

### Pup survival rate after birth

Pregnant *MCH-tTA; TetO DTA* bigenic females (+/+) and control mouse (+/−) were separated from the males and checked once a day for the birth of pups. The number of pups was counted once daily for the first week after birth.

### Target cell activation

Chemogenetics was employed with CNO as a synthetic ligand to activate MCH neurons in *MCH-Cre* mice. To selectively manipulate the MCH neurons, Gq-coupled receptor were used to modify G protein-coupled receptor hM3Dq in a Cre-recombinase-dependent manner. The adeno-associated virus vector, AAV-hSyn-FLEX-hM3Dq-mCherry (hM3-mCherry) (600 nL/injection, 3 × 10^12^ copies/mL) was injected using a 27-gauge stainless steel tube and a push–pull pump injector system (Microprocessor-controlled syringe pumps; World Precision Instruments, Inc., Sarasota, FL, USA) with a diffusion rate of 0.04 mL/min (Bregma: anteroposterior, − 1.5 mm; mediolateral, ± 0.9 mm; dorsoventral, − 5.0 mm) using a stereotactic frame (Devid Kopf Instruments, Tujunga, CA, USA with Stoelting Mouse & Neonatal Rat Adaptor, Wood Dale, IL, USA). The AAV- CMV- FLEX-hrGFP was injected in the same manner as the controls in *MCH-Cre* mice.

For ChR2-mediated MCH cell photoactivation, we used recombinant AAV-EF1a-DIO-hChR2 (E123T/T159C)-EYFP (ChR2-EYFP) vectors that were serotyped with AAV 10 coat proteins and packed by the viral vector core at Jichi Medical University. Cre-dependent ChR2-EYFP AAV vector (AAV-ChR2) (600 nL/injection, 3 × 10^12^ copies/mL) was stereotactically injected into the LHA in the same manner and adjustment (Bregma: anteroposterior, − 1.5 mm; mediolateral, ± 0.9 mm; dorsoventral, − 5.0 mm) described above of virgin *MCH-Cre* female and male mice. All the surgical procedures were performed under anaesthesia using a ketamine-xylazine mixture (100 and 10 mg/kg i.p., respectively). The animals were injected AAV-ChR2 at 8 weeks of age and tested at 20 weeks. The female mice were housed alone starting 1 week before behavioral tests. The male mice were housed alone starting 3 weeks before the tests. The animals were fitted with optic fibre cannulas (diameter 400 μm, length 5 mm) (COME2-FTR/C-F5, LUCIR Inc., Tsukuba, Japan) placed above the LHA bilaterally (AP, L, depth from the brain surface, cannulas were angled 10° toward the outside) or singly for PVN stimulation (AP, L, depth from the brain surface) at least 4 days before the pup presentation test. The optic fibre cable was connected to an optical swivel (COME2-UFC, LUCIR, Inc.) to allow the animals to move around the test cage. Blue laser pulses (473 nm, 10 ms, 0.5 Hz, 1 mW at the tip of the fibre cannula, 400 μm diameter) were administered through the optic fibre during the test sessions. Optogenetic stimulation was performed for the same length of time as the pup presentation (15 min) for mice; for male mice, photo-stimulations were started 5 min before pup presentation. Their behavior was observed for 15 min. The same optogenetic stimulation was performed for GFP-injected females and males. After the pup presentation test, the animals were immediately anaesthetised using isoflurane to collect blood samples from the orbital sinus. The blood samples were drawn into tubes containing EDTA (1 mg/mL blood) and aprotinin (500 KIU/mL of blood, SIGMA). The samples were centrifuged and the supernatants of samples were stored at − 80 °C until hormone assays were performed. The animals were perfused 90 min after the pup-presentation test to obtain brain samples for immunohistochemistry (IHC).

### Open-field assay

Each mouse was placed in the centre of a polypropylene white box (40 × 40 × 30 cm^3^) with approximately 60 lx of illumination for 5 min under video recording. The video was automatically analysed using the Top scan L software (CleverSys, Inc., Reston, Virginia, USA) for the total distance moved.

### Effect of GABA agonists

GABA agonist, muscimol (Sigma, St. Louis, MO) (10 ng) was injected using a 27-gauge stainless steel tube and a push–pull pump (Microprocessor-controlled syringe pumps) with an infusion rate of 0.04 ml/min (Bregma: anteroposterior, − 0.7 mm; mediolateral, ± 0.0 mm; dorsoventral, − 3.5 mm) using a stereotactic frame (Devid Kopf Instruments) into mice. Mice were housed in two groups; one for housing alone and another for continuous group-housing with the same sex siblings separated from litter mates at weaning^4^^.^ The animals were perfused 90 min after injection.

### IHC for MCH, c-fos, oxytocin, orexin, GAD 67 and GABA

All the experimental animals were anaesthetised using an overdose of a pentobarbital sodium–xylazine mixture and perfused with 4% paraformaldehyde. Serial coronal sections (40 µm) were cut using a Microm HM 560 cryostat (Thermo Fisher Scientific, Waltham, MA, USA). For labelling, the sections were incubated overnight with anti-rabbit MCH antibodies (1:10,000, H-070-47, Phoenix Pharmaceuticals Inc. Burlingame, CA, USA), anti-sheep *c-fos* (1:500, AB1584, Millipore Corporation, Temecula, CA, USA, AB1584), anti-rabbit oxytocin (1:2000, PA1-18007, Affinity BioReagents, Golden, CO, USA), PS38 anti-mouse oxytocin monoclonal^45^ (1:500, Gifted from Dr. Higashida), anti-rabbit Orexin A (1:2000, PA1-18310, Thermo Fisher Scientific, Rockford, IL, USA,), anti-guinea pig GABA antibody (1:400, AB175, Sigma), anti-GAD 65, (1:200, sc-32270, Santa Cruz Biotechnology, Inc.) and anti-rat EGFP (1:10,000 dilution, Nakarai Tesque, Inc., Kyoto, Japan, Monoclonal (GF090R)) followed by the secondary antibodies Alexa Fluor 405/488/594-goat anti-rabbit/sheep IgG (Molecular probe, 1:200) and Alexa Fluor 488/594-goat anti-sheep/rat IgG (Molecular probe, 1:200).

### Morphometry

Using the Keyence DC 100 digital imaging system and software (Keyence Microsystems, Osaka, Japan), we digitised images of 200 µm^2^. The number of cells was counted manually using Image J software (National Institutes of Health, Bethesda, Maryland, USA). The expression of AAV-EF1a-DIO-hChR2 (E123T/T159C)-EYFP as an anterograde tracer in MCH neurons allowed us to trace the fibres reaching to the PVN (Fig. 5a) and other brain areas (Supplementary Fig. 7). Images of fibres in the PVN were captured using confocal microscopy (Olympus FV 1200) and LSM 880 Airyscan (Carl Zeiss Co., Ltd.). The section with the largest sectional area was identified, and the area was compared between the animal groups.

### RIA assay

Immediately after performing the behavioral test, blood samples were collected from the orbital sinus of mice anaesthetised with isoflurane. The plasma concentrations of oxytocin were determined using a radioimmunoassay with specific anti-oxytocin, as described previously^46^, after extraction with acetone and diethyl ether. The coefficients of intra-assay and inter-assay variations for oxytocin were 4% and 10%, respectively^47^. The minimum detection limit was 4 pg/mL for oxytocin.

### Statistics

The sample sizes were chosen based on the common practice in animal behavior. Two trained observers blinded to the groups performed all the behavioral scorings. The degree of association between parametric factors was determined using Pearson’s correlation. To compare two behavior groups or number data sets, we used a two-tailed Student’s t-test. When comparing more than two groups, we used repeated two-way ANOVA with post-hoc test of Bonferroni’s method (i.e., for the number of pups in Fig. 1d, the main factors were days after parturition and genotype of the subject). The ratios of behavioral responses in incidence of the categories of maternal behavior in the pup presentation tests were compared using the χ^2^ test with Bonferroni post-hoc tests to examine significant differences. All the statistical analyses were performed using SPSS (version 23, IBM, Armonk, NY, USA). Statistical significance was presented as *p* < 0.05, *p* < 0.01 and *p* < 0.001.

## Supplementary Information

Supplementary Information.

## References

[1] Numan, M. & Insel, T. R. *The Neurobiology of Parental Behavior*. Vol. 1 (Springer Science & Business Media, 2006).

[2] Tachikawa KS, Yoshihara Y, Kuroda KO (2013). Behavioral transition from attack to parenting in male mice: A crucial role of the vomeronasal system. J. Neurosci..

[3] vom Saal FS, Howard LS (1982). The regulation of infanticide and parental behavior: Implications for reproductive success in male mice. Science.

[4] Orikasa C (2015). Social isolation prompts maternal behavior in sexually naïve male ddN mice. Physiol. Behav..

[5] Tsuneoka Y (2013). Functional, anatomical, and neurochemical differentiation of medial preoptic area subregions in relation to maternal behavior in the mouse. J. Comp. Neurol..

[6] Wu Z, Autry AE, Bergan JF, Watabe-Uchida M, Dulac CG (2014). Galanin neurons in the medial preoptic area govern parental behaviour. Nature.

[7] Dulac C, O’Connell LA, Wu Z (2014). Neural control of maternal and paternal behaviors. Science.

[8] Kohl J (2018). Functional circuit architecture underlying parental behaviour. Nature.

[9] Wei YC (2018). Medial preoptic area in mice is capable of mediating sexually dimorphic behaviors regardless of gender. Nat. Commun..

[10] Scott N, Prigge M, Yizhar O, Kimchi T (2015). A sexually dimorphic hypothalamic circuit controls maternal care and oxytocin secretion. Nature.

[11] Marlin BJ, Mitre M, D’amour JA, Chao MV, Froemke RC (2015). Oxytocin enables maternal behaviour by balancing cortical inhibition. Nature.

[12] Nishimori K (1996). Oxytocin is required for nursing but is not essential for parturition or reproductive behavior. Proc. Natl. Acad. Sci. USA..

[13] Bittencourt JC (1992). The melanin-concentrating hormone system of the rat brain: An immuno- and hybridization histochemical characterization. J. Comp. Neurol..

[14] Yao Y, Fu LY, Zhang X, van den Pol AN (2012). Vasopressin and oxytocin excite MCH neurons, but not other lateral hypothalamic GABA neurons. Am. J. Physiol. Regul. Integr. Comp. Physiol..

[15] Qu D (1996). A role for melanin-concentrating hormone in the central regulation of feeding behaviour. Nature.

[16] Shimada M, Tritos NA, Lowell BB, Flier JS, Maratos-Flier E (1998). Mice lacking melanin-concentrating hormone are hypophagic and lean. Nature.

[17] Marsh DJ (2002). Melanin-concentrating hormone 1 receptor-deficient mice are lean, hyperactive, and hyperphagic and have altered metabolism. Proc. Natl. Acad. Sci. USA..

[18] Tsunematsu T (2014). Optogenetic manipulation of activity and temporally controlled cell-specific ablation reveal a role for MCH meurons in sleep/wake regulation. J. Neurosci..

[19] Chung S, Parks GS, Lee CC, Civelli O (2011). Recent updates on the melanin-concentrating hormone (MCH) and its receptor system: Lessons from MCH1R antagonists. J. Mol. Neurosci..

[20] Blouin AM (2013). Human hypocretin and melanin-concentrating hormone levels are linked to emotion and social interaction. Nat. Commun..

[21] Roy M, David N, Cueva M, Giorgetti M (2007). A study of the involvement of melanin-concentrating hormone receptor 1 (MCHR1) in murine models of depression. Biol. Psychiatry.

[22] Sanathara NM (2018). Melanin concentrating hormone modulates oxytocin-mediated marble burying. Neuropharmacology.

[23] Izawa S (2019). REM sleep-active MCH neurons are involved in forgetting hippocampus-dependent memories. Science.

[24] Adams AC (2011). Ablation of the hypothalamic neuropeptide melanin concentrating hormone is associated with behavioral abnormalities that reflect impaired olfactory integration. Behav. Brain Res..

[25] Kokkotou E (2005). Mice with MCH ablation resist diet-induced obesity through strain-specific mechanisms. Am. J. Physiol. Regul. Integr. Comp. Physiol..

[26] Alexander GM (2009). Remote control of neuronal activity in transgenic mice expressing evolved G protein-coupled receptors. Neuron.

[27] Del Cid-Pellitero E, Jones BE (2012). Immunohistochemical evidence for synaptic release of GABA from melanin-concentrating hormone containing varicosities in the locus coeruleus. Neuroscience.

[28] Gies U, Theodosis DT (1994). Synaptic plasticity in the rat supraoptic nucleus during lactation involves GABA innervation and oxytocin neurons: A quantitative immunocytochemical analysis. J. Neurosci..

[29] Adam Y (2019). Voltage imaging and optogenetics reveal behaviour-dependent changes in hippocampal dynamics. Nature.

[30] Saito Y, Cheng M, Leslie FM, Civelli O (2001). Expression of the melanin-concentrating hormone (MCH) receptor mRNA in the rat brain. J. Comp. Neurol..

[31] Li Y, Gao XB, Sakurai T, van den Pol AN (2002). Hypocretin/orexin excites hypocretin neurons via a local glutamate neuron-a potential mechanism for orchestrating the hypothalamic arousal system. Neuron.

[32] Marty A, Llano I (2005). Excitatory effects of GABA in established brain networks. Trends Neurosci..

[33] Kim JS (2011). Chronic hyperosmotic stress converts GABAergic inhibition into excitation in vasopressin and oxytocin neurons in the rat. J. Neurosci..

[34] Lee SW (2015). GABAergic inhibition is weakened or converted into excitation in the oxytocin and vasopressin neurons of the lactating rat. Mol. Brain.

[35] Choi K (2016). Optogenetic activation of septal GABAergic afferents entrains neuronal firing in the medial habenula. Sci. Rep..

[36] Mumtaz F, Khan MI, Zubair M, Dehpour AR (2018). Neurobiology and consequences of social isolation stress in animal model-a comprehensive review. Biomed. Pharmacother..

[37] Matthews GA (2016). Dorsal raphe dopamine neurons represent the experience of social isolation. Cell.

[38] Lonstein JS, Stern JM (1998). Site and behavioral specificity of periaqueductal gray lesions on postpartum sexual, maternal, and aggressive behaviors in rats. Brain Res..

[39] Salzberg HC, Lonstein JS, Stern JM (2002). GABA(A) receptor regulation of kyphotic nursing and female sexual behavior in the caudal ventrolateral periaqueductal gray of postpartum rats. Neuroscience.

[40] Chen PB (2019). Sexually dimorphic control of parenting behavior by the medial amygdala. Cell.

[41] Dölen G, Darvishzadeh A, Huang KW, Malenka RC (2013). Social reward requires coordinated activity of nucleus accumbens oxytocin and serotonin. Nature.

[42] Chung S (2009). The melanin-concentrating hormone system modulates cocaine reward. Proc. Natl Acad. Sci. USA.

[43] Inutsuka A (2014). Concurrent and robust regulation of feeding behaviors and metabolism by orexin neurons. Neuropharmacology.

[44] Inutsuka A (2016). The integrative role of orexin/hypocretin neurons in nociceptive perception and analgesic regulation. Sci. Rep..

[45] Kogami Y (2020). A Monoclonal antibody raised against a synthetic oxytocin peptide stains mouse hypothalamic neurones. J. Neuroendocrinol..

[46] Higuchi T, Uchide K, Honda K, Negoro H (1985). Functional development of the oxytocin release mechanism and its role in the initiation of parturition in the rat. J. Endocrinol..

[47] Onaka T, Yagi K (1990). Differential effects of naloxone on neuroendocrine responses to fear-related emotional stress. Exp. Brain Res..

