# Supplementary material for: Involvement of MCH-oxytocin neural relay within the hypothalamus in murine nursing behavior
Source: Sci Rep. 2021 Feb 8;11:3348. doi: 10.1038/s41598-021-82773-5 (PMC7870840; doi:10.1038/s41598-021-82773-5)
Supplement: Supplementary file 1 — Supplementary Information. [file 41598_2021_82773_MOESM1_ESM.docx]

**Involvement of MCH-oxytocin neural relay within the hypothalamus in murine nursing behavior**

**Yoko Kato^1^, Harumi Katsumata^1^, Ayumu Inutsuka^2^, Akihiro Yamanaka^4^, Tatsushi Onaka^2^, Shiro Minami^1^, Chitose Orikasa^1*^**

**^1^**Department of Bioregulation, Institute for Advanced Medical Science, Nippon Medical School, Kawasaki 211-8533, Japan

**^2^**Department of Physiology, Jichi Medical University, Shimotsuke, Tochigi 329-0498, Japan

**^4^**Department of Neuroscience II, Research Institute of Environmental Medicine, Nagoya University, Nagoya 464- 8601, Japan

**^*^**E-mail: [orikasa@nms.ac.jp](mailto:orikasa@nms.ac.jp)

**Supplementary Figure 1. Generation of *MCH-tTA; TetO DTA* bigenic mice and their reproduction.** (**a-c**) Analysis of female oestrus cycles. Examples of oestrus cycles of *MCH-tTA; TetO DTA* bigenic (+/+) females and control females (+/−) (**a**) for 19 days after their sexual maturations. Number of oestrus (t-test, t = -1.29, df = 14.52, p = 0.22, not significance (NS)) (**b**) and mean period of oestrus (t-test,t = 0.76, df = 13.00, p = 0.46, NS) (**c**). (**d, e**) Latency of mating behaviour from the first mating session. Female mating behaviour with a wildtype (WT) male was observed for 30 mins at their oestrus day (t-test, t = 1.25, df = 17.31, p = 0.23, NS) (**d**). *MCH-tTA; TetO DTA* bigenic and controlfemales gave a birth by similar latency of their mating behaviour session (t-test, t = 0.40, df = 22.50, p = 0.69, NS) (**e**).Data are mean± S.E.M.

**Supplementary Figure 2.** **Ablation of MCH neurons affects**

**decreasing body weight.** Body weight of *MCH-tTA; TetO DTA*

bigenic mice (+/+) and control (+/−) mice at 8 weeks old of age

(t-test, female; t =2.59, df = 15.42, *p = 0.02, male; t = 4.19,

df =19.77, ***p = 0.0005). Data are mean ± S.E.M.

**Supplementary Figure 3. Intruder test of *MCH-tTA; TetO DTA* bigenic male mice.** (**a**) Attack duration,two-way repeated ANOVA, session, F(2,42) = 3.88, *p = 0.028, genotype F(1,42) = 4.96, *p = 0.031,Interaction of session and genotype, F(2,42) = 0.06, p = 0.95, NS. (**b**) Number of biting, two-way repeated ANOVA, session, F(2,42) = 3.38, **p* = 0.044, genotype F(1,42) = 5.09, **p* = 0.029, Interaction of session and genotype, F(2,42) = 0.029, p = 0.97, NS. Data are mean ± S.E.M.

**Supplementary Figure 4. Number of orexin neurons and locomotor activity of MCH neurons ablated and activated virgin female and male mice.** (**a**) Number of MCH and orexin neurons in MCH-Cre mice injected with AAV-DTA (****p*<0.001, t-test) in both sexes. (**b, c**) Locomotor activity measuring the distance moved in a 5 min test in an open arena in females (t-test, t = −0.56, df = 6.45, *p* = 0.60, NS) and males (t-test, t = 0.43, df = 9.53, *p* = 0.67, NS) infected with AAV- DTA and AAV-GFP control (**b**) or infected with AAV-hM3Dq and AAV-GFP control in females (t-test, t = −0.12, df = 8, *p* = 0.991, NS) and males (t-test, t = −0.68, df = 6, *p* = 0.522, NS) (**c**). Data are mean ± S.E.M.

**Supplementary Figure 5. Infection rate, percent of *c-fos***

**expression the LHA**. (**a**) MCH-ir (blue), *c-fos* (red) and ChR2-

EYFP-ir (green) neurons in the LHA, scale bars are 10 µm.

(**b**) Infection rate with ChR2-EYFP expression in the LHA-MCH

neurons. (**c**) Percent of *c-fos* expression in the MCH neurons

labelled with EYFP-ChR2. Data are mean ± S.E.M.

**Supplementary Figure 6. Locomotor activity of virgin female and**

**male mice infected with AAV-ChR2 or AAV-GFP control**. Measuring

the distance moved in a 5 min test in an open arena in the LHA of female

(t-test, t =0.72, df = 12.9, *p* = 0.483, NS) and males (t-test, t =0.94, df = 11,

*p* = 0.367, NS), and in the PVN of females (t-test, t =1.95, df = 9, *p* = 0.084,

NS) and males (t-test, t =1.09, df = 12, *p* = 0.298, NS). Data are mean ± S.E.M.

**Supplementary Figure 7. EYFP-ChR2 fibre distribution in**

**virgin female and male mice injected with AAV-ChR2.**

Projections from MCH neurons are presented in various brain.

Fluorescence ChR2-EYFP labelled projection fibre of mice

with injected ChR2-EYFP-expressing viral vector in the LHA

of MCH-Cre mice. Scales bars are 200 µm in the left and

100 µm in the right. A square indicates place of right figures in

left. LS, lateral septum, DB, diagonal band of broca, MPA,

medial preoptic area.
